# Construction of intracellular asymmetry and asymmetric division in *Escherichia coli*

**DOI:** 10.1038/s41467-021-21135-1

**Published:** 2021-02-09

**Authors:** Da-Wei Lin, Yang Liu, Yue-Qi Lee, Po-Jiun Yang, Chia-Tse Ho, Jui-Chung Hong, Jye-Chian Hsiao, Der-Chien Liao, An-Jou Liang, Tzu-Chiao Hung, Yu-Chuan Chen, Hsiung-Lin Tu, Chao-Ping Hsu, Hsiao-Chun Huang

**Affiliations:** 1 Institute of Molecular and Cellular Biology, National Taiwan University, Taipei, Taiwan; 2 Institute of Chemistry, Academia Sinica, Taipei, Taiwan; 3 Genome and Systems Biology Degree Program, National Taiwan University, Taipei, Taiwan; 4 Department of Life Science, National Taiwan University, Taipei, Taiwan; 5 Graduate Institute of Electronics Engineering, National Taiwan University, Taipei, Taiwan

**Keywords:** Cell division, Cell polarity, Single-cell imaging, Synthetic biology

## Abstract

The design principle of establishing an intracellular protein gradient for asymmetric cell division is a long-standing fundamental question. While the major molecular players and their interactions have been elucidated via genetic approaches, the diversity and redundancy of natural systems complicate the extraction of critical underlying features. Here, we take a synthetic cell biology approach to construct intracellular asymmetry and asymmetric division in *Escherichia coli*, in which division is normally symmetric. We demonstrate that the oligomeric PopZ from *Caulobacter crescentus* can serve as a robust polarized scaffold to functionalize RNA polymerase. Furthermore, by using another oligomeric pole-targeting DivIVA from *Bacillus subtilis*, the newly synthesized protein can be constrained to further establish intracellular asymmetry, leading to asymmetric division and differentiation. Our findings suggest that the coupled oligomerization and restriction in diffusion may be a strategy for generating a spatial gradient for asymmetric cell division.

## Abstract (web summary)

Establishing protein gradients for asymmetric cell division is fundamental across all kingdoms of life. Here the authors construct asymmetric cell division in *E. coli* by localizing the expression of RNA polymerase using an orthogonal unipolar scaffold, and restricting diffusion of its products.

## Introduction

**Fig. 1:**
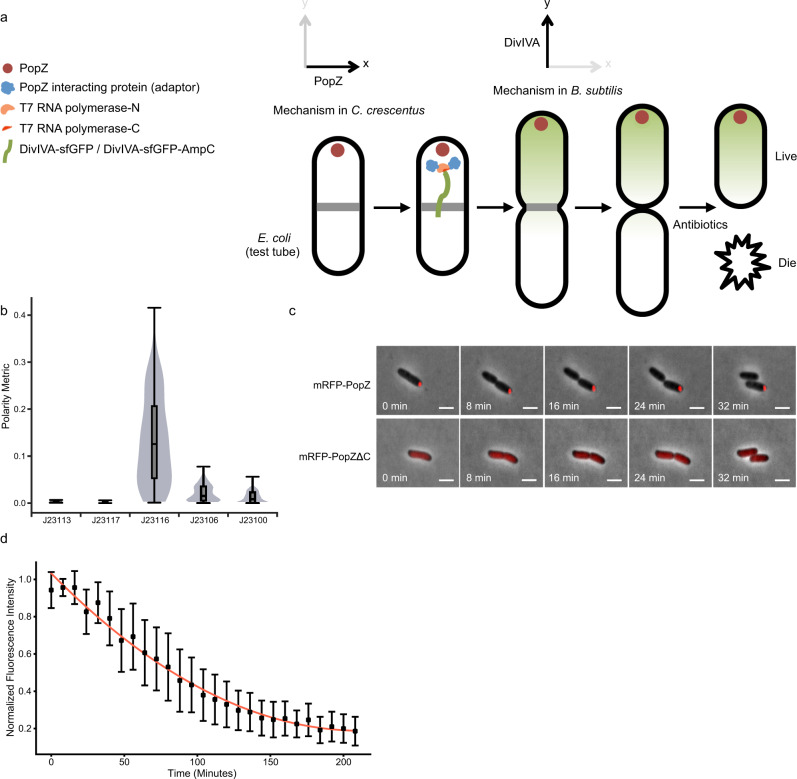
Robust polarity basis for constructing intracellular gradient and asymmetric division in *E. coli*. **a** Graphical illustration of the regulatory layers for asymmetric gene expression and differentiation in *E. coli*. Two orthogonal mechanisms (*x* axis: PopZ from *C. crescentus*; *y* axis: DivIVA from *B. subtilis*) were introduced in *E. coli* to probe the essential features. Split RNA polymerases (N and C halves, shown in light and dark orange) were recruited to PopZ (shown in red) via PopZ-interacting proteins that served as adaptors (shown in blue). The gene expression product, DivIVA-sfGFP or DivIVA-sfGFP-AmpC (shown in green), was restrained from diffusion toward the mid cell and asymmetrically segregated at the first cell division. When two daughter cells were challenged with ampicillin, one cell survived and the other died. **b** Quantification of polarity metric for Anderson promoter series (“Methods”); *n* = 64, 160, 164, 134, and 155 cells for J23113, J23117, J23116, J23106, and J23100, respectively. Center line, median; box limits, upper and lower quartiles; whiskers, 1.5× interquartile range. **c** Time-lapse imaging of cells expressing mRFP-PopZ (top) and mRFP-PopZΔC (bottom) from the Anderson promoter J23116. Scale bars: 1 μm. **d** Quantification of mRFP-PopZ stability. mRFP-PopZ expression was induced from P_*BAD*_ with 2% arabinose for 24 h at 37 °C before time-lapse imaging. Fluorescence intensity was normalized by the maximal intensity. Dots, mean; Error bars, s.d. (*n* = 60 cells). Source data are provided as a Source Data file.

Establishing protein gradients for asymmetric cell division is fundamental for development across all kingdoms of life. In animals, it involves a network of evolutionarily conserved partitioning-defective (PAR) proteins^1,2^, which include scaffolding proteins, adaptors, and enzymes that are asymmetrically enriched in complementary domains^1,2^. Central molecular players and their interactions have been identified and characterized via traditional top–down approaches, and some general principles have begun to emerge. The antagonizing interactions between two distinct domains have been shown to be important for cell polarity^1,2^. Among these interactions, a critical step is the self-oligomerization of scaffold Par-3 (refs. ^3–5^), which is inhibited by the opposing kinase Par-1 (ref. ^6^). It has also been demonstrated that the dynamic association to Par-3 cluster provides PKC-3 kinase with the ability to switch from a localized inactive pool to a diffusive active pool^7–10^. This regulated diffusion allows PKC-3 to shape its activity gradient^7–10^. As the redundancy of these natural interactions complicates the identification of core design principles, synthetic biology presents an engineering approach to reconstruct phenotypes of interest from the ground up^11–13^. Regarding polarity generation, it has been demonstrated in the budding yeast *Saccharomyces cerevisiae* that spontaneous polarization can be constructed using purely synthetic interaction motifs^14^. A recent synthetic study, also carried out in the budding yeast, has demonstrated the minimal PAR network for establishing an opposing polarity gradient^15^. While these studies have provided profound insights, an inherent caveat is the use of yeast Cdc42 as the regulatory node in both aforementioned studies^14,15^ because yeast Cdc42 can still be subject to endogenous regulations such as endocytosis^16,17^. Here, we employ strictly orthogonal mechanisms to perform step-by-step reconstruction of intracellular asymmetry and subsequent asymmetric division in symmetrically dividing *Escherichia coli*. Asymmetric gene expression is chosen as a proof of concept because it is ubiquitous for cell type diversification^18^. Our goal is to probe the design sufficient for RNA polymerase to be asymmetrically functionalized and generate a protein gradient for asymmetric division and differentiation (Fig. 1a).

## Results

### PopZ defines a robust polarity basis

Positive feedback has been suggested to be the core underlying mechanism of cell polarity in both natural^19^ and directly tested synthetic systems^14,20^. As synthetic polarity appears to be unstable and lasts for only tens of minutes when generated solely from feedback interactions^14^, we instead sought natural mechanisms. Interestingly, the central polarity protein PopZ in *Caulobacter crescentus*, an α-proteobacterium that performs asymmetric cell division and differentiation, is known to homo-oligomerize in a similar manner to animal Par-3 (refs. ^21,22^). As *E. coli* lacks a homolog of PopZ, and heterologous expression of PopZ forms foci at a single pole of *E. coli*^21,22^, we tested whether PopZ can provide a robust basis for synthetic cell polarity. We used the medium-level Anderson promoter^23^ J23116 (Supplementary Fig. 1) to express mRFP-PopZ because it achieved the highest score on the polarity metric measuring the sharpness of the pole (Fig. 1b, Supplementary Fig. 2, and “Methods”). The fact that small foci can coalesce into a single focus^21^, and the existence of a switch from unipolar to bipolar when high-level promoters were used (Fig. 1b and Supplementary Fig. 2) suggests that PopZ unipolarity may arise through a similar winner-take-all competition till reaching a saturating concentration^24^. Upon cell division, only one daughter cell inherited the mRFP-PopZ foci (Fig. 1c, top). Meanwhile, fluorescence was diffused throughout and partitioned equally in the oligomerization-domain truncation mutant mRFP-PopZΔC^25^ (Fig. 1c, bottom). To estimate the stability of mRFP-PopZ oligomer in *E. coli*, we measured its fluorescent decay following a transient induction from the arabinose-inducible promoter P_*BAD*_. The mRFP-PopZ persisted for 84 min, roughly 2–3-fold longer than typical *E. coli* division time, before reaching 50% of the initial fluorescence (Fig. 1d and Supplementary Fig. 3). We therefore concluded that oligomeric PopZ can serve as a robust polarized source for synthetic asymmetric cell division in *E. coli*.

### PopZ as a polarized scaffold for asymmetric gene expression

**Fig. 2:**
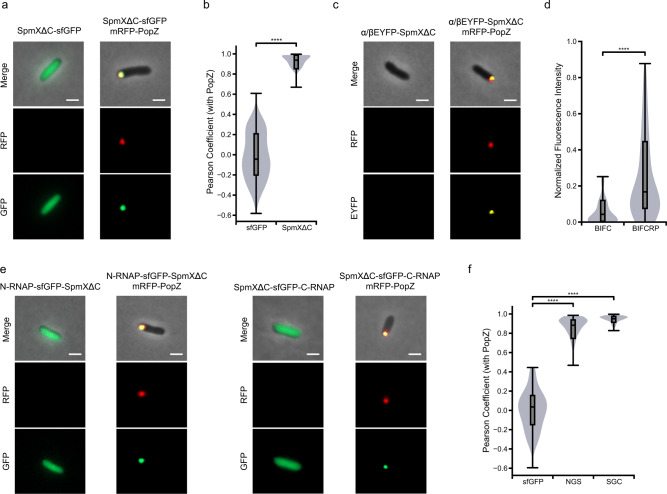
PopZ functions as a polarized scaffold in *E. coli*. **a** Recruitment of SpmXΔC to PopZ. SpmXΔC-sfGFP and mRFP-PopZ were expressed from the Anderson promoters J23113 and J23116, respectively. SpmXΔC-sfGFP was diffused when expressed alone (left) and co-localized with PopZ when co-expressed with mRFP-PopZ (right). Scale bars: 1 μm. **b** Quantification of co-localization with PopZ in **a** (“Methods”). Co-expression of sfGFP and mRFP-PopZ was used as the baseline for comparison (Supplementary Fig. 5); *n* = 82 and 77 cells for sfGFP and SpmXΔC, respectively. Center line, median; box limits, upper and lower quartiles; whiskers, 1.5× interquartile range. Statistical difference was determined by two-tailed Student’s *t*-test; **** denotes *P* < 0.0001. **c** Fluorescence protein complementation via PopZ. The α and β halves of EYFP fused with SpmXΔC were expressed from the Anderson promoter J23117, and mRFP-PopZ was expressed from J23116. Fluorescence was significantly higher when the α and β halves of EYFP fused with SpmXΔC were co-expressed with mRFP-PopZ. Scale bars: 1 μm. **d** Quantification of fluorescence in **c**. BIFCRP and BIFC denote α and β halves of EYFP fused with SpmXΔC were with or without mRFP-PopZ co-expression, respectively; *n* = 125 and 77 cells for BIFCRP and BIFC, respectively. Center line, median; box limits, upper and lower quartiles; whiskers, 1.5× interquartile range (IQR). Fluorescence intensity was normalized by the upper IQR in BIFCRP. Statistical difference was determined by two-tailed Student’s *t*-test; **** denotes *P* < 0.0001. **e** PopZ recruitment of the two halves of PACE-evolved T7 RNA polymerase fused to SpmXΔC. Both SpmXΔC fused PACE-evolved T7 RNA polymerase halves were induced from the P_*tac*_ promoter with 1 mM IPTG for 1 h. mRFP-PopZ was expressed from the Anderson promoter J23116. Scale bars: 1 μm. **f** Quantification of co-localization with PopZ in **e** (“Methods”). NGS and SGC denote RNAP-N-sfGFP-SpmXΔC and SpmXΔC-sfGFP-RNAP-C, respectively. Co-expression of sfGFP and mRFP-PopZ was used as a baseline for comparison (Supplementary Fig. 5); *n* = 83, 118, and 140 cells for sfGFP, NGS, and SGC, respectively. Center line, median; box limits, upper and lower quartiles; whiskers, 1.5× interquartile range. Statistical difference was determined by two-tailed Student’s *t*-test; **** denotes *P* < 0.0001. Source data are provided as a Source Data file.

We next sought to reconstitute asymmetric gene expression using oligomerization-mediated synthetic polarity. Both Par-3 and PopZ are scaffolding proteins that exert polarized functions through direct or indirect (via adaptors) downstream enzyme recruitment^2,26^. We therefore tested whether PopZ can also act as an organizing scaffold when heterologously expressed in *E. coli*. SpmX, a stalk-positioning factor upstream in the *C. crescentus* polarity pathway, was chosen as the adaptor owing to its clear orthogonality (*E. coli* does not have a stalk)^27,28^. SpmX has been shown to be a direct binding partner of PopZ in *E. coli*^26^. A truncation form of SpmX, SpmXΔC, was used (residue 1–150, which contains only the N-terminal lysozyme-like domain^28^; localization of full-length SpmX is shown in Supplementary Fig. 4). SpmXΔC-sfGFP co-localized with mRFP-PopZ when the two proteins were co-expressed, confirming that SpmXΔC contains the region required for the binding of PopZ^28^ (Fig. 2a and b and Supplementary Fig. 5). The ability of PopZ to function as a protein scaffold was tested by fluorescence complementation. The fluorescent protein EYFP was split into α and β halves, and each half was fused with SpmXΔC. Fluorescent foci and increased intensity were only detected when mRFP-PopZ was present (Fig. 2c and d), confirming that PopZ can serve as a docking site to bring split proteins into close proximity and become functional, using SpmXΔC fragments as adaptors. We then questioned whether the two halves of bacteriophage T7 RNA polymerase could be recruited and reassembled at the PopZ hub via SpmXΔC adaptors. A phage-assisted continuous evolution (PACE)-optimized split T7 RNA polymerase was used^29^ because residual affinity was observed between the two halves of the native T7 RNA polymerase^30^ (Supplementary Fig. 6). We confirmed that PopZ could recruit both halves of the T7 RNA polymerase (Fig. 2e and f, simultaneous recruitment of both halves in a single cell is shown in Supplementary Fig. 7).

**Fig. 3:**
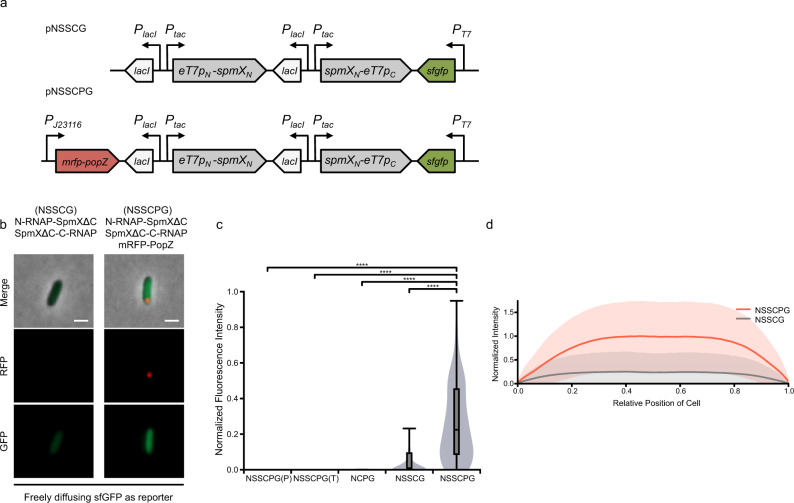
Distribution of freely diffusing reporter remains symmetric. **a** Circuit diagram for asymmetric gene expression when freely diffusing sfGFP was used as a reporter. pNSSCPG and pNSSCG denote plasmids containing SpmXΔC-fused RNAP halves expressed with or without mRFP-PopZ, respectively. *eT7p*_*N*_*-spmX*_*N*_ denotes the SpmXΔC-fused PACE-evolved RNAP-N; *spmX*_*N*_*-eT7p*_*C*_ denotes the SpmXΔC-fused PACE-evolved RNAP-C. Both SpmXΔC-fused T7 RNAP halves were expressed from the IPTG-inducible promoter, P_*tac*_. *sfgfp* is the reporter gene under the expression of T7 promoter. *mrfp-popZ* gene was expressed from the Anderson promoter J23116. **b** Asymmetric gene expression when freely diffusing sfGFP was used as the reporter. NSSCPG and NSSCG denote SpmXΔC-fused RNAP halves expressed with or without mRFP-PopZ, respectively, with sfGFP as the reporter. LAA degradation tag was fused to the C-terminal of sfGFP to reduce background in the control circuit (no PopZ; NSSCG). SpmXΔC-fused RNAP halves were induced from the P_*tac*_ promoter with 50 μM IPTG for 24 h. Fluorescence was significantly higher when SpmXΔC-fused RNAP halves were co-expressed with mRFP-PopZ. Scale bars: 1 μm. **c** Quantification of fluorescence in **b** with three additional negative controls: a Y571S loss-of-function mutation introduced at the C-terminal fragment of T7 RNAP, indicated as NSSCPG(P) (**a**), a truncation (deletion of the 192–883 residues) introduced at the C-terminal fragment of T7 RNAP, indicated as NSSCPG(T) (**b**), and removal of SpmXΔC adaptors from both T7 RNAP halves, indicated as NCPG (**c**). Representative images of **a**–**c** are shown in Supplementary Fig. 8; *n* = 79, 76, 84, 253, and 148 cells for NSSCPG(P), NSSCPG(T), NCPG, NSSCG, and NSSCPG, respectively. Center line, median; box limits, upper and lower quartiles; whiskers, 1.5× interquartile range (IQR). Fluorescence intensity was normalized by the upper IQR in NSSCPG. Statistical difference was determined by two-tailed Student’s *t*-test; **** denotes *P* < 0.0001. **d** Fluorescence intensity (sfGFP) profiles along the long axis of the cell when SpmXΔC-fused RNAP halves were expressed alone (gray lines) or co-expressed with mRFP-PopZ (red lines); *x* = 0 marks the PopZ pole. Fluorescence intensity was normalized by the maximal mean intensity in NSSCPG. Solid lines indicate averages; colored belts indicate standard deviations; *n* = 148 and 147 cells for with and without mRFP-PopZ co-expression, respectively. Source data are provided as a Source Data file.

Using sfGFP as the reporter for the gene of interest driven by the T7 promoter, fluorescence intensity was found to increase significantly when SpmXΔC-fused split T7 RNA polymerase was co-expressed with PopZ, indicating that RNA polymerase was reassembled and functional (Fig. 3a–c). We confirmed that the presence of PopZ did not promote sfGFP expression when a loss-of-function mutation (Y-571S)^31^ or truncation was introduced to the C-terminal fragment of the T7 RNA polymerase (Fig. 3c and Supplementary Fig. 8). PopZ also did not induce the expression of sfGFP when SpmXΔC adaptors were absent (Fig. 3c and Supplementary Fig. 8). However, for freely diffusing sfGFP, there appeared to be no intracellular fluorescence gradient (Fig. 3d), nor any difference in the fluorescence between the two recently divided daughter cells (Supplementary Fig. 9). A diffusible sfGFP produced from a localized source with a diffusion constant of ~7 μm^2^ s^−1^ (measured in an earlier^32^ and also this study) would spread over thousands of micrometers after half an hour, thus was unlikely to establish any gradient in an *E. coli* cell.

### A second orthogonal layer to circumvent diffusion

**Fig. 4:**
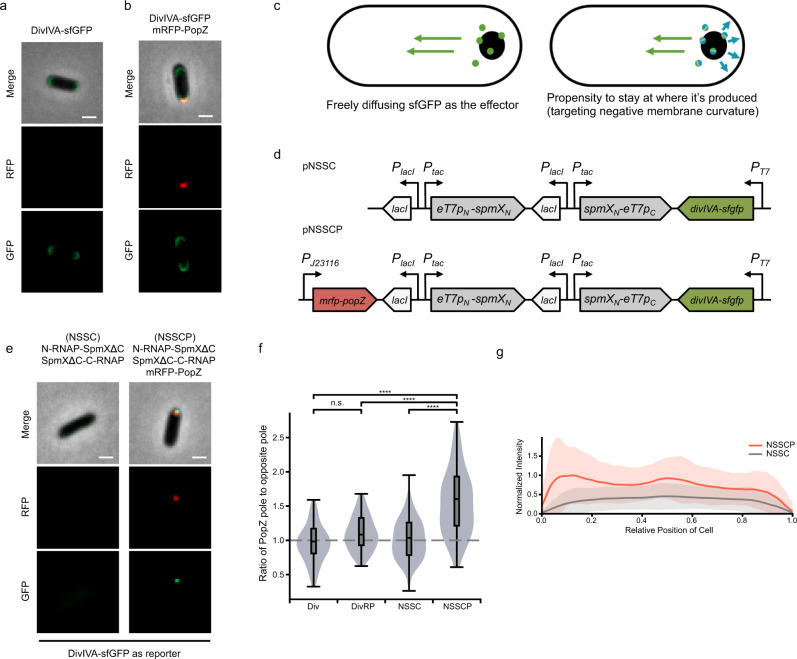
Intracellular asymmetry arises when DivIVA is used as a reporter. **a** Localization of DivIVA-sfGFP when the expression was induced from the P_*tac*_ promoter with 50 μM IPTG for 1 h. Scale bars: 1 μm. **b** Localization of DivIVA-sfGFP and mRFP-PopZ when the two were co-expressed. DivIVA-sfGFP expression was induced from the P_*tac*_ promoter with 50 μM IPTG for 1 h. mRFP-PopZ expression was induced from the P_*BAD*_ promoter with 2% arabinose for 2 h. Scale bars: 1 μm. **c** Graphic comparing freely diffusing GFP and limited diffusion via DivIVA. **d** Circuit diagram for asymmetric gene expression when DivIVA-sfGFP was used as the reporter. pNSSCP and pNSSC denote plasmids containing SpmXΔC-fused RNAP halves expressed with or without mRFP-PopZ, respectively. *eT7p*_*N*_*-spmX*_*N*_ denotes the SpmXΔC-fused PACE-evolved RNAP-N; *spmX*_*N*_*-eT7p*_*C*_ denotes the SpmXΔC-fused PACE-evolved RNAP-C. Both SpmXΔC-fused T7 RNAP halves were expressed from the IPTG-inducible promoter, P_*tac*_. *divIVA-sfgfp* is the reporter gene under the expression of T7 promoter. *mrfp-popZ* gene was expressed from the Anderson promoter J23116. **e** Asymmetric gene expression when DivIVA-sfGFP was used as a reporter. NSSCP and NSSC denote SpmXΔC-fused RNAP halves expressed with or without mRFP-PopZ, respectively, with DivIVA-sfGFP as a reporter. SpmXΔC-fused RNAP halves were induced from the P_*tac*_ promoter with 50 μM IPTG for 1 h. Scale bars: 1 μm. **f** Quantification of DivIVA-sfGFP asymmetry in **a**, **b**, and **e** using the ratio between opposite poles (“Methods”). NSSCP and NSSC denote SpmXΔC-fused RNAP halves expressed with or without mRFP-PopZ, respectively. Div denotes DivIVA-sfGFP expressed alone. DivRP denotes DivIVA-sfGFP co-expressed with mRFP-PopZ. The gray dashed line indicates one (i.e. no asymmetry); *n* = 89, 78, 99, and 91 cells for Div, DivRP, NSSC, and NSSCP, respectively. Center line, median; box limits, upper and lower quartiles; whiskers, 1.5× interquartile range. Statistical difference was determined by two-tailed Student’s *t*-test; **** denotes *P* < 0.0001. **g** Fluorescence intensity (sfGFP) profiles along the long axis of the cell when NSSC circuits were expressed alone (gray lines) or co-expressed with mRFP-PopZ (red lines); *x* = 0 marks the PopZ pole. Fluorescence intensity was normalized by the maximal mean intensity in NSSCP. Solid lines indicate averages; colored belts indicate standard deviations; *n* = 99 and 91 cells for NSSC and NSSCP, respectively. Source data are provided as a Source Data file.

Nature has evolved a variety of mechanisms for circumventing the protein diffusion to ensure asymmetric cell division across species. Among these are physical barriers, such as the bud neck in *Saccharomyces*^33,34^, and spore septum in *Bacillus subtilis*^35^. When there is no physical compartmentalization, diffusive protein mobility must be actively countered. This occurs through mechanisms such as directed transport^36^ or slow membrane diffusion/endocytosis in Cdc42 (ref. ^17^), diffusion state switching in MEX-5/PIE-1 (ref. ^37^), or regulated association with anchoring structures in Par-3/PKC-3 (refs. ^7–10^). It has also recently been reported that PopZ can create a micro-domain to sequester its downstream signaling molecules to shape the transcriptional gradient^38^. To independently address the effect of restricting diffusion, we employed a mechanism orthogonal to PopZ oligomerization. DivIVA is another oligomeric protein from *B. subtilis* with a propensity to target negative membrane curvature^39,40^. It can target division sites and cell poles in *E. coli*^41,42^ and has been used as a polar anchor^26^. We confirmed that when expressed alone under a constitutive promoter, DivIVA-sfGFP localized at both poles without apparent asymmetry (Fig. 4a, f). As the semipermeable PopZ complex has been shown to allow membrane-associated proteins to explore the pole and enter into the membrane proximal to PopZ^38^, we next asked whether DivIVA-sfGFP, known to target the inner membrane via its hydrophobic residues at the N terminus^43^, can still recognize the two poles and remain bipolar when mRFP-PopZ is present. Both mRFP-PopZ and DivIVA-sfGFP maintained unipolar and bipolar localization, respectively, when the two were co-expressed (Fig. 4b, f), supporting that PopZ does not have a significant effect on prohibiting DivIVA for membrane targeting and that the two natural mechanisms are orthogonal. We reasoned that when DivIVA-sfGFP is used as the reporter for asymmetrically localized T7 RNA polymerase, it would have a higher tendency to target the pole at which it has been produced, as opposed to diffusing freely in the cytoplasm (Fig. 4c and d). In addition, according to Stokes-Einstein law, we expected DivIVA to have a smaller diffusion coefficient given its oligomeric nature^39^. The degree of asymmetry was quantified using constitutively expressed DivIVA-sfGFP as the baseline (“Methods”, Fig. 4a, f). When SpmXΔC-fused split T7 RNA polymerase was co-expressed with PopZ, there was a significant increase in DivIVA-sfGFP asymmetry, with higher intensity at the PopZ pole (Fig. 4d–g).

**Fig. 5:**
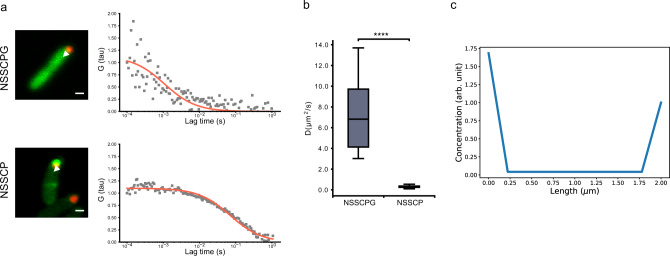
Diffusion measurement and modeling confirm that asymmetry can arise with DivIVA as a reporter. **a** Representative images and FCS curves for NSSCPG (measuring freely diffusing sfGFP, top) and NSSCP (measuring DivIVA-sfGFP, bottom). Measurements were taken after SpmXΔC-fused RNAP halves were induced from the P_*tac*_ promoter with 50 μM IPTG for 1 h. The white triangles indicate the locations for FCS measurements. In both plots, gray dots are the measured data points, whereas the solid orange lines indicate the fitted functions. Scale bars: 1 μm. **b** Mobility of the protein products. Diffusion coefficients were calculated from the fit *τ*_D_ (“Methods”) and the values were 7.23 ± 3.05 μm^2^ s^−1^ (*n* = 24) and 0.32 ± 0.1 μm^2^ s^−1^ (*n* = 23) for NSSCPG (freely diffusing sfGFP) and NSSCP (DivIVA-sfGFP), respectively. Center line, median; box limits, upper and lower quartiles; whiskers, 1.5× interquartile range. Statistical difference was determined by two-tailed Student’s *t*-test; **** denotes *P* < 0.0001. **c** Modeling results recapitulated the observed asymmetry (ratio between opposite poles: 1.68). This was aimed to recapitulate the averaged value for NSSCP in Fig. 4f; *x* = 0 marks the PopZ pole (localized source of DivIVA production). Source data are provided as a Source Data file.

Next, measurement using fluorescence correlation spectroscopy (FCS) confirmed that the diffusion coefficient is smaller in DivIVA-sfGFP (Fig. 5a and b and Supplementary Fig. 10). This measured diffusion coefficient was then incorporated into a one-dimensional reaction-diffusion model that describes DivIVA oligomer production, diffusion, and cooperative membrane association (Supplementary Note 1; cell growth has been omitted for simplicity), and the model recapitulated the observed asymmetry (Fig. 5c, with an opposite ratio of 1.68 that recapitulated the averaged asymmetry in NSSCP circuit shown in Fig. 4f; the model was also able to achieve an opposite ratio of over 4 as observed experimentally; see Supplementary Note 1 for parameter scans). While full-length SpmX has been reported to oligomerize^44^, SpmXΔC alone (fragment containing the lysozyme-like domain^28^) was present mostly as a monomer with a minor population that formed oligomers^44^, suggesting only weak self-interaction. To test whether PopZ can still functionalize the circuit without the potential SpmXΔC self-association, we replaced one of the SpmXΔC adaptors with CpdR, an adaptor for ClpXP protease in *C. crescentus*^45^ and also a PopZ direct binding protein^26^. We confirmed that CpdR co-localized with PopZ when PopZ was present (Supplementary Fig. 11). When the SpmXΔC/CpdR pair was used as an adaptor for split T7 RNA polymerase (Supplementary Fig. 12a), background fluorescence was significantly reduced (Supplementary Fig. 12c), and the degree of asymmetry was still apparent (Supplementary Fig. 12b, d, e), although a higher IPTG induction was necessary for the activation (20-fold higher than when dual SpmXΔC was used as the adaptor, Supplementary Fig. 12). Altogether, these data demonstrated that in addition to PopZ defining a localized source for generating a rapidly diffusing pool of downstream effector proteins, a restriction in diffusion via another oligomeric, pole-targeting DivIVA is necessary for establishing asymmetry at the length scale of an *E. coli* cell.

### Asymmetric division and differentiation in *E. coli*

**Fig. 6:**
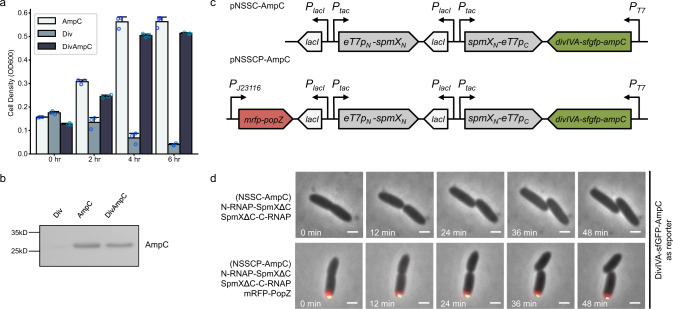
Use of beta-lactamase as a cell fate determinant. **a** DivIVA-sfGFP-AmpC conferred resistance to ampicillin at a level that is closed to sfGFP-AmpC. DivIVA-sfGFP (Div), sfGFP-AmpC (AmpC), and DivIVA-sfGFP-AmpC (DivAmpC) were expressed from the Anderson promoter J23106; 100 μg mL^−1^ ampicillin was added to the liquid culture for the indicated times and cell growth was measured by optical density using plate reader (“Methods”). Data are presented as means ± s.d. (*n* = 3 experiments). **b** DivIVA-sfGFP-AmpC can secrete the matured beta-lactamase to periplasm as sfGFP-AmpC. DivIVA-sfGFP (Div), sfGFP-AmpC (AmpC), and DivIVA-sfGFP-AmpC (DivAmpC) were expressed from the Anderson promoter J23106. Periplasmic extract was collected, and secreted beta-lactamase was detected by western blotting using the anti-AmpC antibody (“Methods”). **c** Circuit diagram for asymmetric gene expression when DivIVA-sfGFP-AmpC was used as the reporter, and SpmXΔC was used as the adaptor for both halves of RNAP. pNSSCP and pNSSC denote plasmids containing SpmXΔC-fused RNAP halves expressed with or without mRFP-PopZ, respectively. *eT7p*_*N*_*-spmX*_*N*_ denotes the SpmXΔC-fused PACE-evolved RNAP-N; *spmX*_*N*_*-eT7p*_*C*_ denotes the SpmXΔC-fused PACE-evolved RNAP-C. Both SpmXΔC-fused T7 RNAP halves were expressed from the IPTG-inducible promoter, P_*tac*_. *divIVA-sfgfp-ampC* is the reporter gene under the expression of T7 promoter. *mrfp-popZ* gene was expressed from the Anderson promoter J23116. **d** Time-lapse imaging of SpmXΔC-fused RNAP halves expressed with (NSSCP-AmpC, bottom) or without (NSCC-AmpC, top) mRFP-PopZ, with DivIVA-sfGFP-AmpC as a reporter. The AAV degradation tag was fused to the C-terminal of DivIVA-sfGFP-AmpC to reduce the leakage in the absence of IPTG (i.e. basal resistance to ampicillin). SpmXΔC-fused RNAP halves were induced from the P_*tac*_ promoter with 50 μM IPTG for 3 h. Scale bars: 1 μm. Source data are provided as a Source Data file.

**Fig. 7:**
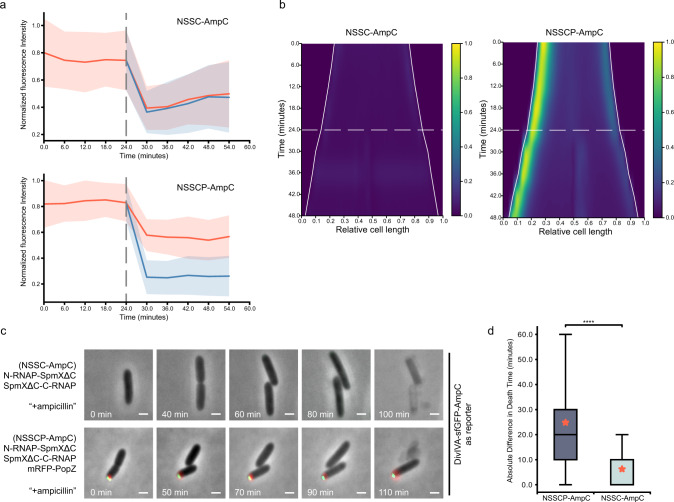
Asymmetric division of cell fate determinant and differentiation in *E. coli*. **a** Quantification of total fluorescence within mother (before cell division) and two daughter cells (after cell division) as shown in Fig. 6d. NSSCP-AmpC (bottom) and NSSC-AmpC (top) denote circuits with or without mRFP-PopZ, respectively. Fluorescence intensity was normalized by the maximal intensity in each cell. The gray dashed lines indicate the time of cell division. If mRFP-PopZ is present in the genetic circuit, red and blue denote daughter cells with or without the inheritance of mRFP-PopZ, respectively. If there is no PopZ in the circuit, the two daughters will be randomly assigned to red or blue groups. Solid lines indicate averages; colored belts indicate standard deviations; *n* = 60 and 61 cells for NSSC-AmpC and NSSCP-AmpC, respectively. **b** Kymographs of DivIVA-sfGFP-AmpC along the long axis of the cell over time. NSSCP-AmpC (right) and NSSC-AmpC (left) denote circuits with or without mRFP-PopZ, respectively. Fluorescence intensity of NSSC-AmpC was normalized to NSSCP-AmpC. PopZ is located at *x* = 0. White solid lines indicate cell boundaries; white dashed lines indicate the time points of cell division; *n* = 60 and 61 cells for NSSC-AmpC and NSSCP-AmpC, respectively. **c** Time-lapse imaging when NSSC-AmpC (top) and NSSCP-AmpC (bottom) were challenged with ampicillin; 100 mg mL^−1^ ampicillin was added onto the agarose pad as the challenge after the mother cell fully divided into two daughter cells (at *t* = 60 min and 50 min for NSSC-AmpC and NSSCP-AmpC, respectively). Death was defined at the time when a cell underwent membrane rupture/lysis^50^. Scale bars: 1 μm. **d** Absolute difference in death time between sister cells upon ampicillin treatment for NSSC-AmpC and NSSCP-Amp. Center line, median; box limits, upper and lower quartiles; whiskers, 1.5× interquartile range. Red asterisks indicate averages (6.25 min and 24.83 min for NSSC-AmpC and NSSCP-AmpC, respectively); *n* = 64 and 61 cells for NSSC-AmpC and NSSCP-AmpC, respectively. Statistical difference was determined by two-tailed Student’s *t*-test; **** denotes *P* < 0.0001. Source data are provided as a Source Data file.

Lastly, we investigated whether the asymmetric division of cell fate determinants and functional differentiation can be achieved with this design. Beta-lactamase (AmpC), an enzyme that can confer resistance to ampicillin antibiotics, was fused with DivIVA-sfGFP to serve as a proof of concept because the innate properties of beta-lactamase could allow further processing and secretion to the periplasm^46–48^ (i.e., our preferred direction, Fig. 4c), as opposed to diffusing back toward the mid-cell. We confirmed that DivIVA-sfGFP-AmpC conferred resistance to ampicillin at a level that was comparable to sfGFP-AmpC (Fig. 6a; normal growth without ampicillin is shown in Supplementary Fig. 13a). The matured form of beta-lactamase could also be detected in the periplasm for DivIVA-sfGFP-AmpC (Fig. 6b; purity of the fractionation is shown in Supplementary Fig. 13b). Next, the asymmetrically reassembled T7 RNA polymerase was used to express DivIVA-sfGFP-AmpC (Fig. 6c). We introduced a C-terminal SsrA degradation tag (AAV^49^) to the AmpC fusion to lower the basal resistance from the T7 promoter leakage (Supplementary Fig. 14). Upon induction, the daughter cell that inherited the mRFP-PopZ foci inherited significantly more green fluorescence at the first cell division (Figs. 6d and  7a). The asymmetric inheritance was also evident in the kymograph that contains spatial information (Fig. 7b). When the two recently divided daughter cells were challenged with ampicillin, the daughter with mRFP-PopZ showed an increase in survival time (Fig. 7c) which was evident from a significantly higher absolute time difference in death as compared to the control (Fig. 7d, death is defined at the time of membrane rupture/lysis^50^), indicating diversification of cell fates. This was in contrast to the case when sfGFP-AmpC was equally divided into the two daughter cells (Supplementary Fig. 15a–c). When ampicillin was added as a challenge, both daughter cells survived until the end of the time-lapse experiment (i.e., we observed no difference in death time for all pairs of sister cells by the end of the imaging, *n* = 68 cell divisions; Supplementary Fig. 15d).

## Discussion

The cell-pole organizing protein PopZ was first identified as a multimeric protein that acts as a hub to polarize functions in *Caulobacter*^21,22^. When heterologously expressed in *E. coli*, PopZ remains as a functional scaffold to recruit its binding partners^21,22^, and this polar recruitment can be adopted as a useful platform for detecting protein–protein interactions, for example^51^. An asymmetric division of the PopZ scaffold in *E. coli* has been reported since its first identification^21^. Only recently did a study begin to directly couple PopZ to downstream signaling for a synthetic cell differentiation mechanism in *E. coli*^52^. Specifically, a c-di-GMP-degrading phosphodiesterase was fused with PopZ to differentiate c-di-GMP levels, and differential gene expression was achieved via the asymmetric inheritance of this key enzyme^52^. Note that in the study, differentiation (i.e., the difference in fluorescent reporter levels) was observed at the second or later divisions^52^. Here, we demonstrated that imposing a constraint on diffusion could generate an intracellular gradient for effector proteins, such that now alternative cell fates were observed at the first cell division. From a synthetic biology perspective, the ability to create an intracellular gradient and differentiate cells at the first cell division would allow synthetic morphogenesis^53^ with bit sizes precisely at single cells, thus presenting another important step forward. As high-copy plasmids also tend to localize at nucleoid-free regions^54,55^ and were used in this study, T7 promoters that are present on plasmids should be accessible to the polarly localized T7 RNA polymerase. If the reporter constructs were to be chromosomally encoded, another layer of polar recruitment of the chromosome via the ParS/ParB system^56^ would allow co-localization with the polarized T7 RNA polymerase (for example, by sandwiching the T7 promoters between two ParS arrays).

Using two orthogonal oligomeric proteins, PopZ and DivIVA, we successfully reconstructed intracellular asymmetry and subsequent asymmetric division from the bottom up. We showed that PopZ defines a robust polarity, and DivIVA acts to restrict the diffusion of downstream effector proteins. We demonstrated that when expressed moderately, the PopZ micro-domain appeared permeable to DivIVA for membrane targeting and did not prohibit the formation of the usual DivIVA scaffold (Fig. 4b, f). However, spatial occlusion between PopZ and DivIVA can be anticipated when both scaffolding proteins grow into larger oligomeric structures, and in such cases, it is plausible that the microdomain formed by PopZ (and the adaptors as added layer) would act as another restriction to the movement of DivIVA toward mid-cell. Taken together, evidence from our study supports that limiting diffusion may be an important underlying physical constraint for maintaining an intracellular spatial gradient universal for prokaryotes^38^ and eukaryotes^7–10,17,36,37^. Mutual inhibition was dispensable in our system, but it can be anticipated that an antagonizing signal at the other pole should further sharpen the gradient/increase the robustness of the differentiation^14,57^.

## Methods

### Bacterial strains and growth conditions

DH5α, the recombination-deficient (*recA1* deletion) strain of *E. coli*, was used throughout the study. The genotype is as follows: *end*A1 *rec*A1 *rel*A1 *gyr*A96 *hsd*R17(*rk*^*−*^*, mk*^*+*^) *pho*A *sup*E44 *thi*-1 Δ(*lac*ZYA-*arg*F)U169 Φ80 Δ(*lac*Z)M15 F^−^.

Cells were grown overnight at 28 °C in LB media with antibiotics. Cultures were subsequently diluted (dilution factor: 20-fold) and grown in 4 mL of LB media until they reached an optical density of 0.3–0.4 at 600 nm, followed by induction or sampling for microscopy. Thirty-five micrograms per milliliter chloramphenicol was used for cloning and plasmid maintenance; 100 μg mL^−1^ ampicillin was used for the plate reader experiment; 100 mg mL^−1^ ampicillin was added onto the agarose pad for the death–survival drug challenge experiment for the observation of rapid lysis under basal resistance.

### Plasmid circuit construction

Circuits in this study were constructed mainly using BioBrick standard assembly^58^. Briefly, DNA fragments encoding proteins and promoters were standardized with flanking cloning sites (prefix: EcoRI and XbaI; suffix: SpeI and PstI) and could be subsequently combined to create larger circuits with unified protocol. Protein fusions were generated mainly using circular polymerase extension cloning (CPEC)^59^. Point mutations were introduced by site-directed mutagenesis. All circuits used in this study were cloned into the high-copy-number plasmid pSB1C3 as single plasmids. All circuits (between prefix and suffix) in this study were sequenced by Sanger sequencing (Genomics, Taiwan). The plasmid names, major features, the complete circuit DNA sequences have been listed in Supplementary Data 1; major primers have been listed in Supplementary Data 2.

The PopZ coding sequence was isolated from the genome of *C. crescentus*. mRFP was fused to the N-terminus of PopZ, as its C-terminus contains the oligomerization domain^25^. The mRFP-PopZ fusion was subsequently combined with P_*BAD*_ or Anderson promoter^23^ BioBricks for quantitative expression.

The SpmX coding sequence was isolated from the genome of *C. crescentus*. The 1–150 residues (N-terminal lysozyme-like domain)^28^ were amplified from full-length SpmX as SpmXΔC. Fluorescent proteins (EYFP or sfGFP) were fused to the C-terminus of SpmXΔC for localization analysis. For fluorescence complementation, YFP was split at residue 158 (ref. ^60^), and SpmXΔC was fused at the C-terminus of both α (1–158 residue) and β (159–241 residue) halves. These SpmXΔC fusions were subsequently combined with the Anderson promoter^23^ BioBricks for expression.

Fragments of native T7 RNA polymerase and T7 promoter^30^ were purchased from Addgene. PACE-evolved split T7 RNA polymerase^29^ was a kind gift from Dr. Bryan Dickinson (University of Chicago, USA). SpmXΔC was fused to the C-terminal and N-terminal of the N and C fragments of the T7 RNA polymerase, respectively. Fluorescent proteins (sfGFP or EBFP2) were sandwiched between SpmXΔC and T7 RNA polymerase fragment for localization analysis. These SpmXΔC-fused T7 RNA polymerase fragments were subsequently combined with the P_*tac*_ promoter BioBrick (containing one copy of LacI) for inducible expression. For T7 RNA polymerase loss-of-function, the substitution of Serine for Tyrosine at position 571 (ref. ^31^) and deletion of the 192–883 residues were introduced as point mutation and truncation, respectively, to the C-terminal fragment.

The DivIVA coding sequence was isolated from the genome of *B. subtilis*. sfGFP was fused to the C-terminus of DivIVA and subsequently combined with T7 or Anderson promoter^23^ BioBricks for expression. AmpC was amplified from the pSB1A3 plasmid and used as C-terminal fusion with DivIVA-sfGFP.

### Microscopy

Cells were sandwiched between agarose pads (by dissolving 1% agarose in distilled water and LB for snapshot and time-lapse experiments, respectively, and prepared as previously described^61^) and 8-well μ-Slide (iBidi) for imaging. Phase contrast and epifluorescent images were acquired using a Zeiss AxioObserver Z1 inverted microscope equipped with an EC Plan-Neofluar 100× objective/1.30NA (Carl Zeiss), a Coolsnap HQ2 camera (Photometrics), and an X-Cite 120Q (Excelitas) excitation light source. The microscope was operated by Zen software. Snapshot and time-lapse experiments were performed at 28 °C and 30 °C, respectively, in a humidified chamber unless otherwise specified.

### Quantification of fluorescent images

Outlines and fluorescence intensity profiles of individual cells were extracted from cell lists obtained from Oufti^62^, and subjected to customized analysis programs written in Python. Fluorescence intensity was calculated by the total fluorescence normalized by the length of the cell. The degree of co-localization was quantified by Pearson’s correlation coefficient between two fluorescent channels. Quantitative analysis of PopZ polarity and the degree of asymmetry are detailed below.

### Analysis of PopZ polarization

As the overexpression of mRFP-PopZ led to congested/bipolar patterns (Supplementary Fig. 2), we quantified the pole sharpness with a polarity metric defined as the maximal value divided by the width of the PopZ signal. We selected the expression level of mRFP-PopZ based on this metric. The maximal intensity value was extracted from Oufti^62^. To measure pole width, the fluorescence intensity profile along the long axis of the cell was subject to automatic image thresholding using Otsu’s method. This converted the fluorescence profile into a one-dimensional Boolean vector containing only 0’s (below threshold) and 1’s (above threshold). The width was calculated as the fraction of 1’s in that vector. Only cells with sharp PopZ unipolar foci (consecutive 1’s occupying less than 1/3 of the vector) were chosen for subsequent analysis.

### Quantification of the degree of asymmetry

The fluorescence intensity profile along the long axis of the cell was extracted from Oufti and can be used as a statistical visualization of symmetry/asymmetry. If PopZ is present in the genetic circuit, fluorescent images will be oriented such that PopZ locates at the same position (*x* = 0). If there is no PopZ in the circuit, the two poles will be randomly assigned (*x* = 0 or *x* = 1). The degree of asymmetry in DivIVA-GFP was quantified by calculating the ratio between opposite poles, defined as the sum of the fluorescence intensity at one pole (1/3 cell length) divided by the sum of the fluorescence intensity at the other pole (1/3 cell length).

### FCS measurement

FCS measurements were carried out using a time-resolved laser scanning confocal nanoscope (Q2; ISS Inc.). The system is set up on an inverted Nikon Eclipse Ti microscope equipped with a water immersion objective (Plan Apo 60X; N.A. = 1.2, Nikon) and a 488 nm laser Diode (NDS4116; Nichia Corp.) as the excitation source. The fluorescence emitted from the sample was collected by a GaAs photomultiplier tube detector (H7422P-40; Hamamatsu) through a bandpass filter (505–545 nm; Semrock Inc.). The output of the PMT unit was recorded via the time-correlated single photon counting (TCSPC) data acquisition module. The diameter of detection pinhole was set to 50 μm, and typical laser power for the measurement was set to 1 μW. Meanwhile, the beam waist radius (*ω*_0_) and the structural parameter (*S*) necessary for the FCS analysis were determined by calibrating using the aqueous Rhodamine 110 solution with reported diffusion coefficient of 440 μm^2^ s^−1^ before each experiment^63^. We thus obtained the values of 0.26 μm and 9 for *ω*_0_ and *S*, respectively, and such values were used for subsequent FCS analysis.

To measure the diffusion coefficients of samples, cells carrying proteins of interest were prepared on a Nunc Lab-Tek II chambered coverglass (ThermoFisher Scientific) before mounting onto the microscope. FCS measurements were performed at room temperature for 120 s for each cell. Cells with similar expression levels of GFP fluorescence were selected, and TCSPC data recording intensity fluctuations were binned and collected at the frequency of 1 μs. For each sample, the autocorrelation function (ACF) was obtained by averaging ACF from at least 4 × 12 s intensity traces. Any ACF curves showing large fluctuations were assumed to reflect large vesicles or fluorescent aggregates and thus were excluded for the calculation^64^. The resulting ACF curves were then fitted using a simple one-component 3D diffusion model to obtain diffusion coefficients using the ISS VistaVision software (ISS Inc.).

### Periplasmic extraction and western blotting analysis

Cell pellet from the 10-mL culture with an optical density of 2.0 at 600 nm was washed with 850 μL of PBS (NaCl 137 mM, KCl 2.7 mM, Na_2_HPO_4_ 24.2 mM, KH_2_PO_4_ 5.2 mM, pH 7.4), and the suspension was centrifuged at 21,000 × *g* at 4 °C for 3 min. Thereafter, the supernatant obtained was discarded, and the pellet was resuspended in 900 μL of spheroplast buffer (0.1 M Tris pH 8.0, 500 mM sucrose, 0.5 mM EDTA pH 8.0); this was incubated for 5 min and centrifuged, after which the supernatant obtained was discarded, and the pellet was resuspended in 400 μL of hypotonic solution (1 mM MgCl_2_). After a 15-s incubation on ice followed by the addition of 20 μL of MgSO_4_ (20 mM), the cell suspension was centrifuged, and the supernatant was transferred into a fresh tube as the periplasmic fraction. Cell pellet was transferred into another tube as the cytoplasmic fraction. The purity of fractionation was confirmed with a cytoplasmic sfGFP using plate reader to measure fluorescence (Supplementary Fig. 13b).

A 15-μL aliquot of the periplasmic fraction was separated on a 10% gel using SDS-PAGE and transferred onto PVDF blotting membranes (PolyScreen PVDF Hybridization Transfer Membrane; PerkinElmer). The membranes were washed in PBS with 0.05% Tween 20 (PBST). After blocking with a gelatin-NET buffer for 1 h at room temperature, the membranes were incubated with an anti-ampC antibody (1:5000 dilution; cat. no. MBS310846; MyBioSource, Inc.) overnight at 4 °C. After incubation with the corresponding HRP-conjugated secondary antibody (anti-mouse; 1:5000 dilution; cat. no. NEF822001EA; PerkinElmer) for 1 h at room temperature, immunoreactive proteins were detected using an enhanced chemiluminescence detection system (Luminol Reagent Plus and Oxidizer Reagent Plus).

### Optical density and fluorescence measurements

Cell densities, based on absorbance, were measured using a microplate reader (SpectraMax i3x Multi-Mode Microplate Reader) with a SpectraPlate-96 MB, 96-well microplate and 200 µL per well. Absorbance was measured at a wavelength of 600 nm at room temperature. To set up blanks for measurements, 200 μL of liquid LB medium was added to a blank well without inoculation. For the measurement of fluorescence in cell fractionation, a black-wall 96-well microplate (Interlab, Inc.) was used; Tris buffer was used to set up blanks.

### Statistics and reproducibility

All statistical analyses were performed in Python. Two-tailed *t*-test was used to determine differences between two groups. *P* < 0.05 was considered to indicate a statistically significant difference. All experiments were performed with at least three biologically independent samples.

### Reporting summary

Further information on research design is available in the Nature Research Reporting Summary linked to this article.

## Supplementary information

Supplementary Information

Reporting Summary

Description of Additional Supplementary Files

Supplementary Data 1

Supplementary Data 2

## Data Availability

All data and materials from this work are available from the corresponding author upon reasonable request. Source data are provided with this paper.

## Source data

Source Data

## Code availability

The custom code for polarity analysis is available at https://github.com/dwgoblue/SynPolarityAnalysis (10.5281/zenodo.4297656); modeling code is available at https://github.com/cherrihsu/asymmetrycells (10.5281/zenodo.4305098).
