# Supplementary material for: Construction of intracellular asymmetry and asymmetric division in Escherichia coli
Source: Nat Commun. 2021 Feb 9;12:888. doi: 10.1038/s41467-021-21135-1 (PMC7873278; doi:10.1038/s41467-021-21135-1)
Supplement: Supplementary file 1 — Supplementary Information [file 41467_2021_21135_MOESM1_ESM.pdf]

Supporting Information for

"Construction of intracellular asymmetry and asymmetric division in Escherichia coli"

Da-Wei Lin[1, 6], Yang Liu[1, 6], Yue-Qi Lee[1], Po-Jiun Yang[1], Chia-Tse Ho[1], Jui-Chung Hong[1], Jye-Chiah Hsiao[2], Der-Chien Liao[1], An-Jou Liang[1], Tzu-Chiao Hung[1], Yu-Chuan Chen[2], Hsiung-Lin Tu[2, 3], Chao-Ping Hsu[2, 3] and Hsiao-Chun Huang*[1, 3-5].

[1]Institute of Molecular and Cellular Biology, National Taiwan University, Taipei 10617, Taiwan, [2]Institute of Chemistry, Academia Sinica, Taipei 11529, Taiwan, [3]Genome and Systems Biology Degree Program, National Taiwan University, Taipei 10617, Taiwan, [4]Department of Life Science, National Taiwan University, Taipei 10617, Taiwan, [5]Graduate Institute of Electronics Engineering, National Taiwan University, Taipei 10617, Taiwan, [6]These authors contributed equally to this work.

*Correspondence: hsiaochun@ntu.edu.tw (H.-C.H.)

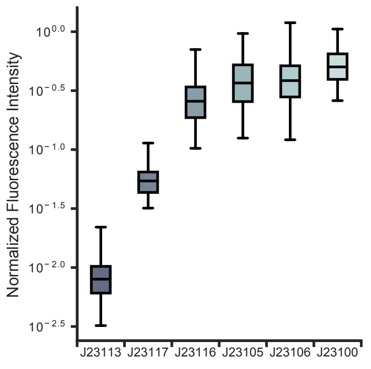

Supplementary Figure 1. Quantification of Anderson promoter strengths with mWasabi as reporter. Center line, median; box limits, upper and lower quartiles; whiskers, 1.5x interquartile range (IQR). Fluorescence intensity was normalized by the upper IQR in J23100. n=136, 139, 112, 137, 166, 97 cells for J23113, J23117, J23116, J23105, J23106 and J23100, respectively.

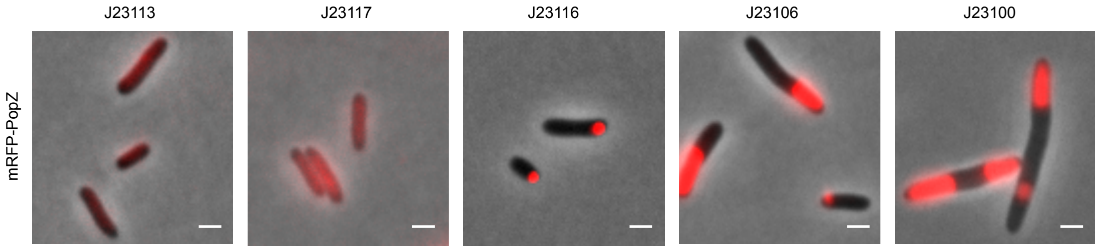

Supplementary Figure 2. Images of mRFP-PopZ expressed from indicated Anderson promoters. Scale bars: 1 μm.

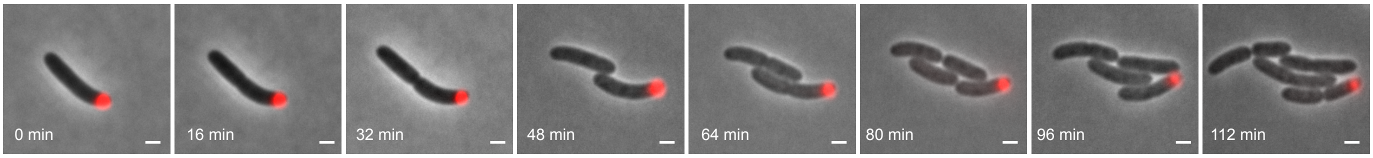

Supplementary Figure 3. Time-lapse images of mRFP-PopZ induced from pBAD. Scale bars: 1 µm.

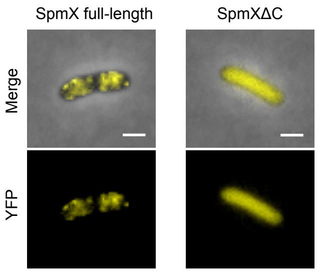

Supplementary Figure 4. mages of full-length (left) and truncated (right) SpmX. Both were expressed under J23117 promoters. Scale bars: 1 μm.

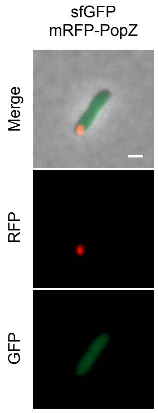

Supplementary Figure 5. Localization of sfGPP when co-expressed with mRFP-PopZ. sfGFP and mRFP-PopZ were expressed under J23106 and J23116 promoter, respectively. Scale bars: 1 μm.

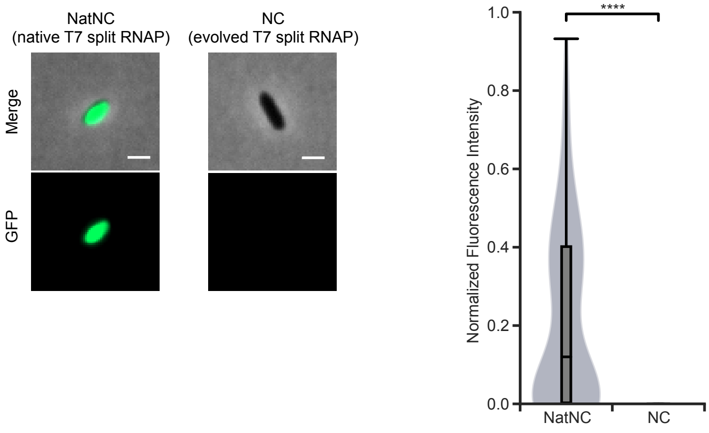

Supplementary Figure 6. Comparison of background fluorescence between native (NatNC) and PACE-evolved (NC) split RNA polymerase. Both were expressed under Tac promoter without IPTG induction (i.e. measuring leakage). Scale bars: 1 µm. Center line, median; box limits, upper and lower quartiles; whiskers, 1.5x interquartile range (IQR). Fluorescence intensity was normalized by the upper IQR in NatNC. n=106 and 83 cells for NatNC and NC, respectively. Statistical difference was determined by two-tailed Student's *t*-test. **** denotes $P < 0.0001$.

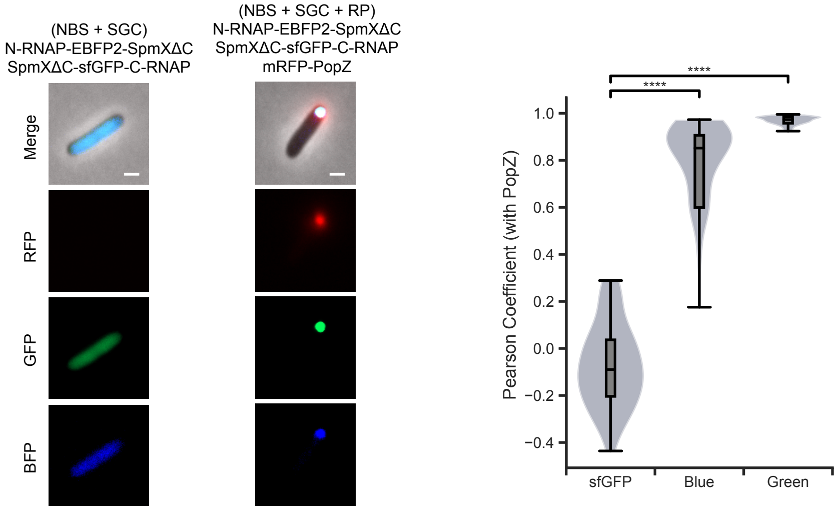

Supplementary Figure 7. PopZ recruitment of both halves of PACE-evolved T7 RNA polymerase fused to SpmXΔC expressed in the same cells. (Right) NBS and SGC denote RNAP-N-EBFP2-SpmXΔC and SpmXΔC-sfGFP-RNAP-C, respectively. RP denotes mRFP-PopZ. NBS and SGC were induced from pTac promoter with 1 mM IPTG for 1 h. mRFP-PopZ was expressed from the Anderson promoter J23116. All three fluorescent fusions were expressed in a single cell. Imaging was performed at 37 °C for a better blue fluorescence. Scale bars: 1 µm. (Left) Quantification of co-localization with PopZ for NBS (from blue fluorescence channel, indicated as blue) and SGC (from green fluorescence channel, indicated as green) in the same cells (Methods). Co-expression of sfGFP and mRFP-PopZ was used as a baseline for comparison (also performed at 37 °C). n= 84 and 78 cells for sfGFP and NBS+SGC, respectively. Center line, median; box limits, upper and lower quartiles; whiskers, 1.5x interquartile range. Statistical difference was determined by two-tailed Student's *t*-test. **** denotes $P < 0.0001$.

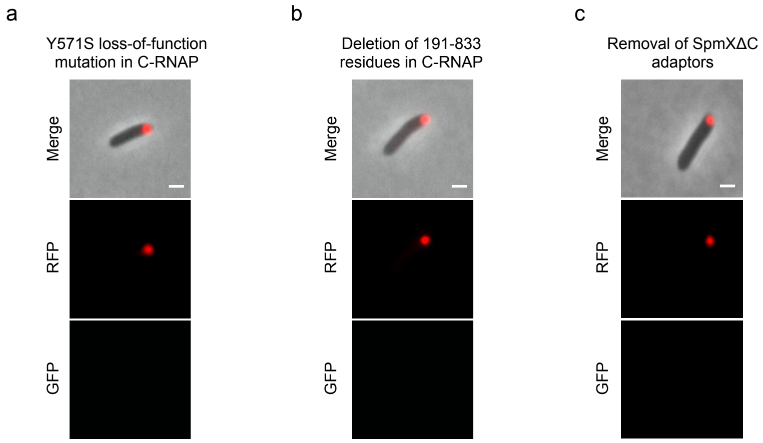

Supplementary Figure 8. Representative images of three negative controls for NSSCPG: (a) a Y571S loss-of-function mutation introduced to the C-terminal fragment of T7 RNAP, (b) a truncation (deletion of the 191-883 residues) introduced to the C-terminal fragment of T7 RNAP, and (c) removal of SpmXΔC adaptors from both T7 RNAP halves. All circuits were induced from the pTac promoter with 50 μM IPTG for 24 h. Scale bars: 1 μm.

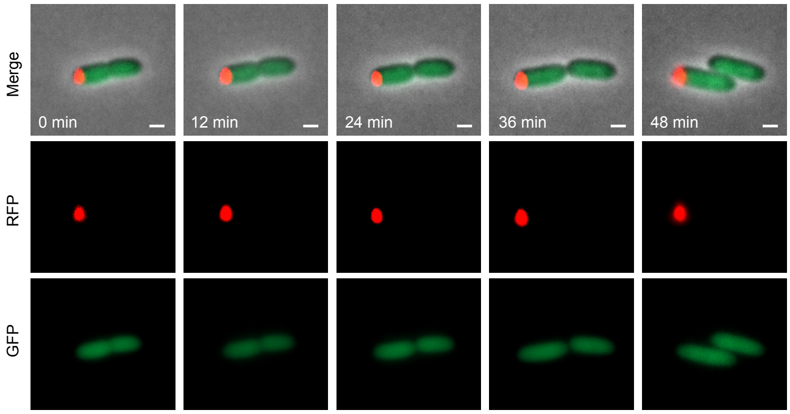

Supplementary Figure 9. Representative images of a dividing cell when SpmXΔC-fused RNAP halves were co-expressed with mRFP-PopZ and freely diffusing sfGFP was used as a reporter. SpmXΔC-fused RNAP halves were induced from the pTac promoter with 50 μM IPTG for 1 h. Scale bars: 1 μm.

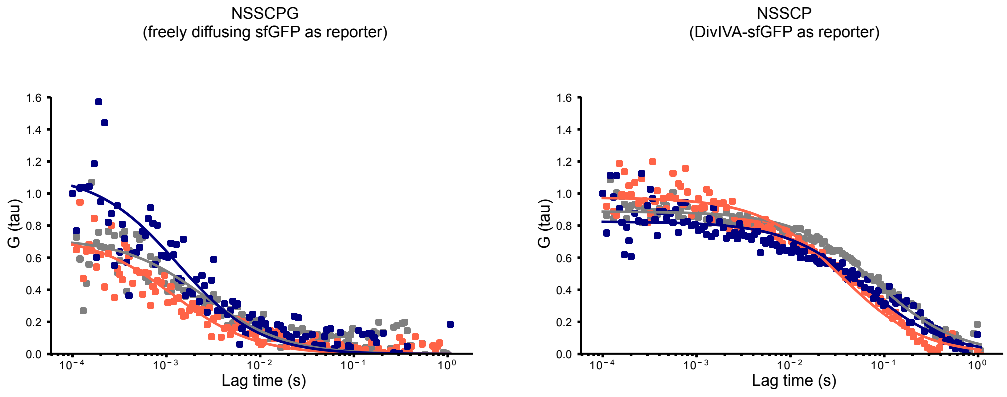

Supplementary Figure 10. Additional representative FCS curves for NSSCPG (measuring freely diffusing sfGFP, left) and NSSCP (measuring DivIVA-sfGFP, right). Measurements were taken after SpmXΔC-fused RNAP halves were induced from the pTac promoter with 50 μM IPTG for 1 h. In both plots, colored dots are the measured data points, whereas the solid colored lines indicate fitted functions.

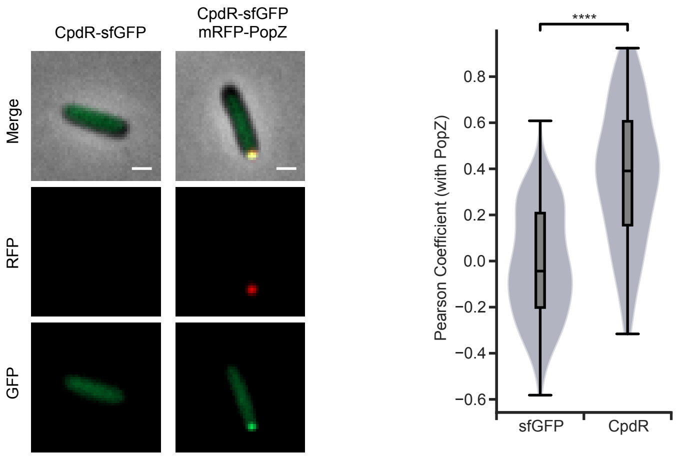

Supplementary Figure 11. Recruitment of CdpR to PopZ. CpdR-sfGFP and mRFP-PopZ were expressed from the Anderson promoter J23100 and J23116, respectively. CpdR-sfGFP was diffused when expressed alone and co-localized with PopZ when co-expressed with mRFP-PopZ. Scale bars: 1 μm. Quantification of co-localization with PopZ is shown on the right (Methods). Co-expression of sfGFP and mRFP-PopZ was used as the baseline for comparison (Supplementary Fig. 5; used as in Figure 2b for direct comparison). n= 82 and 152 cells for sfGFP and CpdR, respectively. Center line, median; box limits, upper and lower quartiles; whiskers, 1.5x interquartile range. Statistical difference was determined by two-tailed Student's *t*-test. **** denotes P < 0.0001.

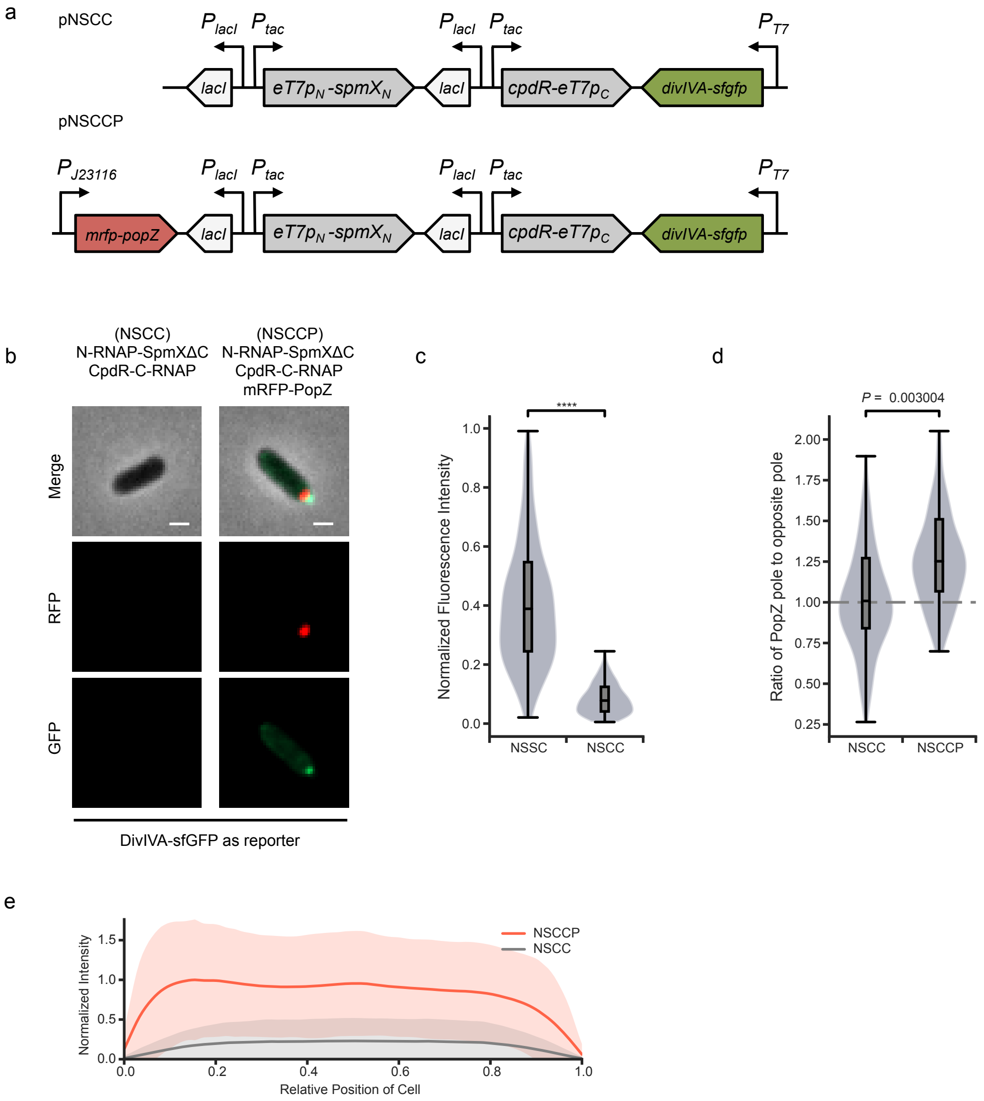

Supplementary Figure 12. Asymmetric gene expression when DivIVA-sfGFP was used as a reporter and one of the adaptors was replaced with CpdR. (a) Circuit diagram. SpmXΔC and CpdR were used as the adaptors for the N- and C-terminal of PACE-evolved RNAP, respectively. pNSCCP and pNSCC denote plasmids with or without mRFP-PopZ, respectively. *eT7pN-spmXc* denotes the SpmXΔC fused PACE-evolved RNAP-N; *cpdR-eT7pc* denotes the CpdR fused PACE-evolved RNAP-C. Both SpmXΔC-fused RNAP-N and CpdR-fused RNAP-C were expressed from the IPTG-inducible promoter, pTac. *divIVA-sfgfp* is the reporter gene under the expression of T7 promoter. *mrfp-popZ* gene was expressed from the Anderson promoter J23116. (b) Asymmetric gene expression when DivIVA-sfGFP was used as the reporter, and one of the adaptors was replaced with CpdR. NSCCP and NSCC denote with or without mRFP-PopZ, respectively. SpmXΔC/CpdR-fused RNAP halves were induced from the pTac promoter with 1 mM IPTG for 1 h. Fluorescence asymmetry was apparent when SpmXΔC/CpdR-fused RNAP halves were co-expressed with mRFP-PopZ. Scale bars: 1 µm. (c) Reduction of background fluorescence when comparing NSSC and NSCC. NSSC (dual SpmXΔC) and NSCC (SpmXΔC/CpdR pair) denote the background of RNAP halves expressed without mRFP-PopZ. n=180 and 143 cells for NSSC and NSCC, respectively. Center line, median; box limits, upper and lower quartiles; whiskers, 1.5x interquartile range (IQR). Fluorescence intensity was normalized by the upper IQR in NSSC. Statistical difference was determined by two-tailed Student's *t*-test. **** denotes P < 0.0001. (d) Quantification of DivIVA-sfGFP asymmetry in (b) using the ratio between opposite poles (Methods). NSCCP and NSCC denote SpmXΔC/CpdR-fused RNAP halves expressed with or without mRFP-PopZ, respectively. The gray dashed line indicates one (i.e., no asymmetry). n=122 and 105 cells for NSCC and NSCCP, respectively. Center line, median; box limits, upper and lower quartiles; whiskers, 1.5x interquartile range. Statistical difference was determined by two-tailed Student's *t*-test. (e) Fluorescence intensity profiles along the long axis of the cell when NSCC circuits were expressed alone (gray lines) or co-expressed with mRFP-PopZ (red lines). Fluorescence intensity was normalized by the maximal mean intensity in NSCCP. Solid lines indicate averages; colored belts indicate standard deviations. n=141and 200 cells for NSCC and NSCCP, respectively.

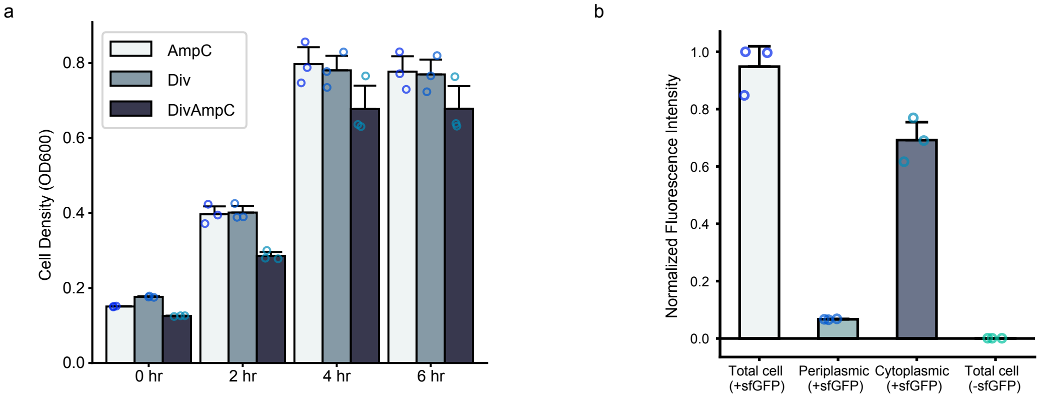

Supplementary Figure 13. DivIVA-sfGFP-AmpC can secrete the matured beta-lactamase to periplasm and confer resistance to ampicillin as sfGFP-AmpC. (a) Normal growth (i.e. without ampicillin) of cells expressing DivIVA-sfGFP (Div), sfGFP-AmpC (AmpC) and DivIVA-sfGFP-AmpC (DivAmpC) from the Anderson promoter J23106 measured at the indicated times by optical density using plate reader (Methods). (b) Purity of periplasmic fraction. Cytoplasmic sfGFP expressed from the Anderson promoter J23106 was used to confirm whether the inner membranes remain intact after the preparation of periplasmic fraction. 2-mL culture with an optical density of 2.0 at 600 nm was used for fractionation as described in the Methods. Total cell lysate (i.e. cytoplasmic + periplasmic; sonication was used for cell lysis) with and without the expression of sfGFP were used as positive and negative controls, respectively. 200 µL out of 1mL lysate volume was taken for fluorescence measurement using plate reader (Methods). Data are presented as means ± s.d. (n = 3 experiments). The majority of fluorescence remained in the cytoplasmic fraction, suggesting that the preparation of periplasmic fraction did not disrupt the inner membrane to cause severe contamination.

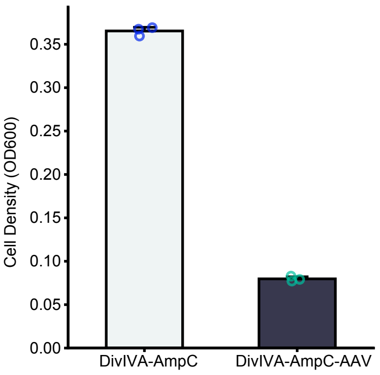

Supplementary Figure 14. Comparison of basal resistance (without IPTG induction) between NSSC circuits using DivIVA-AmpC and DivIVA-AmpC-AAV (AAV degradation tag fused to C-terminus of AmpC) as reporters; 100 µg mL-1 ampicillin was added to the liquid culture for 12 h before cell growth was measured by optical density using plate reader (Methods). Data are presented as means ± s.d. (n = 3 experiments).

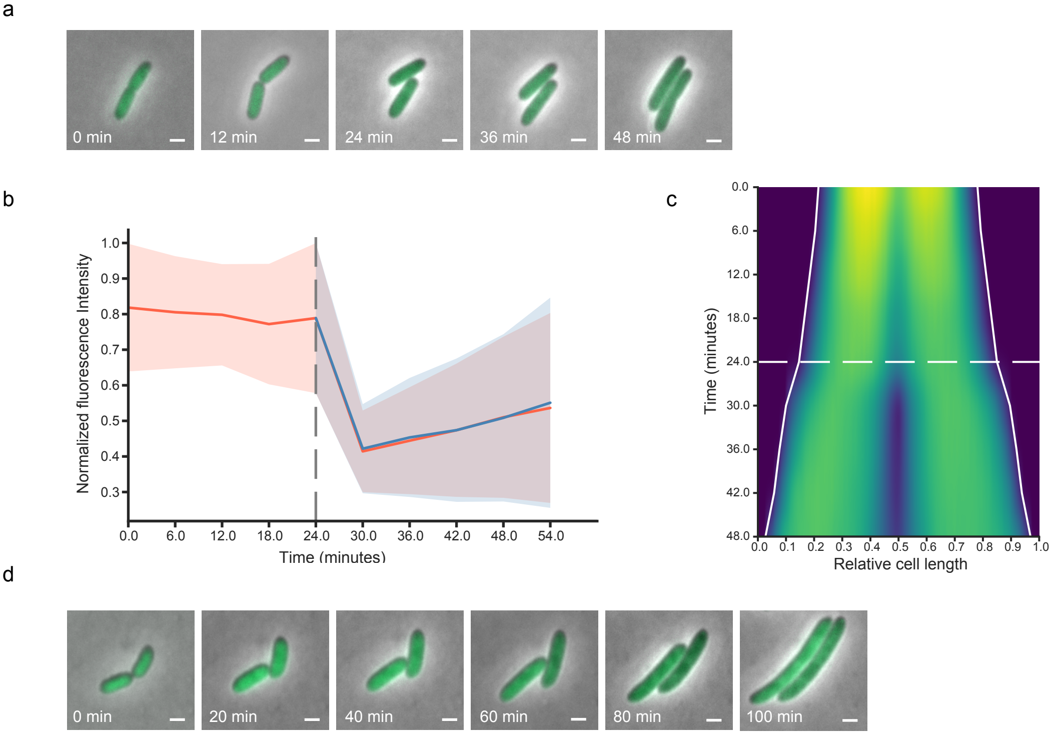

Supplementary Figure 15. Similar response to ampicillin between two daughter cells when sfGFP-AmpC was diffused and divided equally. sfGFP-AmpC was expressed from the Anderson promoter J23106. (a) A representative time-series image (normal cell division without ampicillin as the challenge). Scale bars: 1 μm. (b) Quantification of the total fluorescence within mother (before cell division) and two daughter cells (after cell division) as shown in (a). The gray dashed lines indicate the time of cell division. Solid lines indicate averages; colored belts indicate standard deviations. (c) Kymographs of sfGFP-AmpC along the long axis of the cell over time. White solid lines indicate cell boundaries; white dash lines indicate the times of cell division. n = 60 cells analyzed in (b) and (c). (d) A representative time-series images when challenged with ampicillin; 100 mg mL-1 ampicillin was added onto the agarose pad as the challenge after the mother cell fully divided into two daughter cells (at t = 20 min). Scale bars: 1 μm.

Supplementary Note 1

Numerical simulation

We defined a one-dimensional mathematical model to describe the production and membrane binding for DivIVA. The membrane-bound form was denoted as m, and freely diffusible form, u.

$$\frac{\partial u}{\partial t} = D\nabla^2 u + k_p - k_d u - \left[u k_b\big(\delta + (1-\delta)H(m)\big) - k_s m\right] \ , \quad (1)$$

$$\frac{\partial m}{\partial t} = u k_b(\delta + (1-\delta)H(m)) - k_s m \ , \quad (2)$$

where $H(m)$ is the Hill's function for the synergistic effects of membrane binding,

$$H(m) = \frac{m^n}{K^n + m^n} \ .$$

In Eqs (1) and (2), D is the diffusion coefficient, production and degradation rates of the free form DivIVA are k_p and k_d, respectively. k_b is the total, maximum membrane binding rate, k_s is the corresponding rate for DivIVA to dissociate from the membrane. We have assumed that the membrane binding is cooperative (with the Hill's function shown above). δ is the fraction of initial, non-cooperative membrane binding. We further restricted the production of u to be at one end of *E. coli*, and the membrane binding is allowed only at the two poles[1,2]. In our numerical simulation, we simplified the problem to an array of 1-dimensional grids, with a modest 10 grids in total. k_p was set to zero except for the first grid where PopZ is localized. Similarly, k_b is nonzero only at the first and the last grids. In this model, m is not diffusible, and it only exists at the two ends.

We further noted that DivIVA is known to oligomerize[3]. With the measured diffusion constant 0.32 $\mu m^2/s$ for DivIVA-sfGFP (Fig. 5b), which is 20 times smaller than that of GFP, we can assume that the diffusible component is already oligomerize in the cytosol[4] and the concentration of such oligomers are modeled as u, and m. The membrane binding may further enhance recruitment of DivIVA[2], and thus a Hill's function was used to describe the synergy.

The diffusion in Eq (1) was solved using a simple finite difference scheme, and the time evolution was propagated using the Euler's explicit method. The parameters employed are as listed in Supplementary Table 1, which was set under a reasonable consideration for the amount and dynamics of protein observed before[4]. The parameters were determined with the following considerations. First, we noted that the steady-state concentration determines the scale of the concentration, i.e., if there were no diffusion, the steady-state value for u would have been,

$$u_{ss} = \frac{k_p}{k_d},$$

and for m, we estimated the "maximum steady-state value" allowed for m by setting the Hill's function to its maximum value 1, which is

$$m_{ss}^{max} = \frac{k_b u_{ss}}{k_s} = \frac{k_b k_p}{k_s k_d}.$$

We also noted that the time scales of the dynamics are essentially determined by the degradation and the dissociation rates k_d and k_s.[5] Therefore, while keeping the expected (maximum) steady-state concentrations at reasonable levels (u_{ss} = 60 nM and m_{ss}^{max} = 3000 nM), we scanned for the degradation and the dissociation rates k_d and k_s (Supplementary Fig. 16), and the asymmetric ratio is defined as the ratio of total amount of DivIVA-sfGFP oligomer (u+m) in the first grid, to that in the last grid. We have also scanned u_{ss} and m_{ss}^{max} (by varying k_p and k_b while keeping k_d and k_s fixed) and the results are in Supplementary Fig. 17. Supplementary Table 1 lists the final parameters that we employed for Fig. 5c in the main text.

By assuming a nonlinear, cooperative membrane binding, there could be a bistablility for a low- and a high-level of m, and the transition between them is hysteretic and slow[6]. Therefore, the unipolar build-up of m is achieved by a membrane binding at one pole that is fast enough, but it has not reached the high-concentration state yet at the other pole, and this can be achieved by the lag in the slow, hysteretic dynamics in membrane binding. As seen in Supplementary Fig. 16, a unipolar region at the upper region where membrane disassociation (k_s) is fast, with a range of k_d that somehow matches. If k_d, the degradation rate for u, is too fast, it stops m from building up in both ends, and a slow k_d makes bipolar cells. Results in Supplementary Fig. 17 can be understood similarly. A high asymmetry ratio is observed in the high m_{ss}^{max} region, where the capacity of membrane binding (with a high membrane association rate, k_b) is high. If u_{ss} is too high, the system ends with a bipolar situation. A low u_{ss} leads to insufficient membrane binding at both ends. Therefore, the mathematical model reproduced the unipolar buildup of DivIVA through the different membrane binding kinetics at both ends, which is a result of cooperative binding.

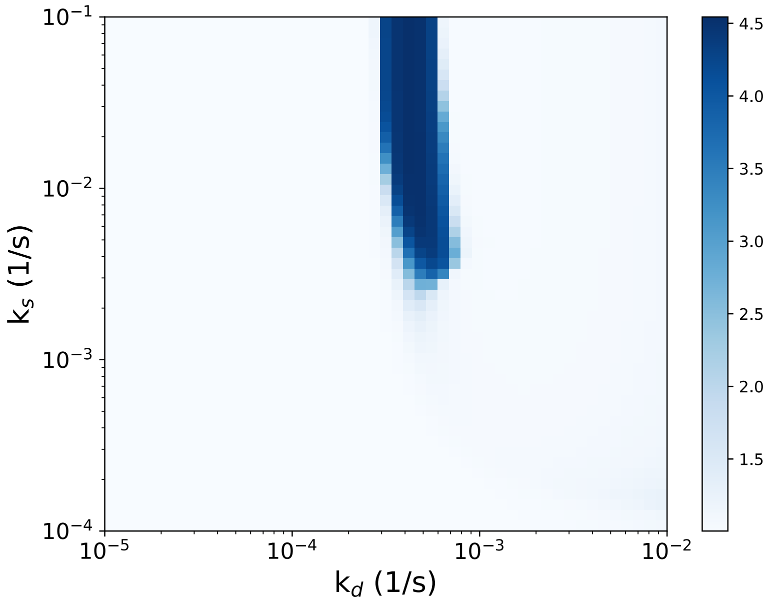

Supplementary Figure 16. The asymmetry ratio, the amount of membrane-bound DivIVA (m) in the first grid divided by that in the last grid, with different parameters k_s and k_d. k_p and k_s values were scaled to keep u_{ss} and m_{ss}^{max} values fixed at 60 and 3000 nM. Other settings are as listed in Supplementary Table 1.

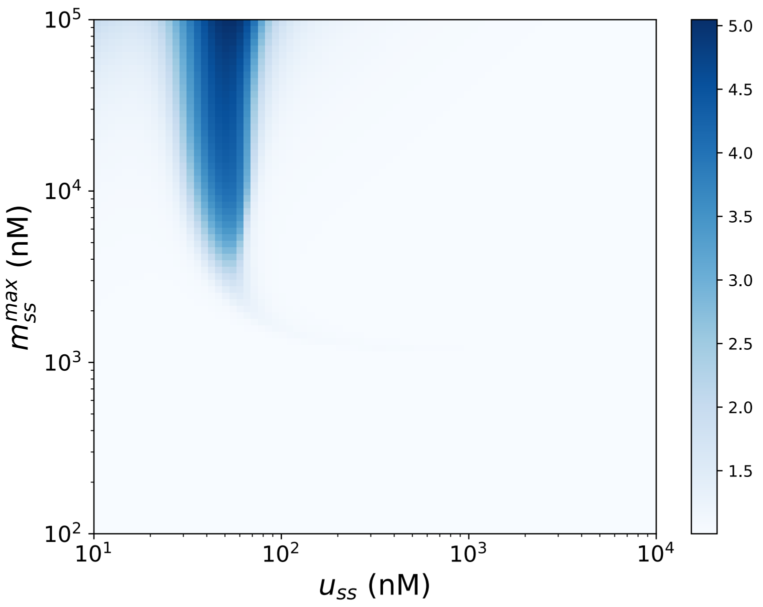

Supplementary Figure 17. The asymmetry ratio with different (maximum) steady-state values u_{ss} and m_{ss}^{max} by varying k_p and k_b, while keeping k_d and k_s fixed at 0.0005 and 0.0024 1/s. Other settings are as listed in Supplementary Table 1.

Supplementary Table 1. Parameters used for the data presented.

**PARAMETER**	**VALUE**	**UNITS**	**NOTES**
D	0.32	µm^2/s	As measured
k_d	0.0005	1/s	
u_{ss}	60	nM	Corresponds to k_p = 0.03 nM/s
k_s	0.0024	1/s	
m_{ss}^{max}	3000	nM	Corresponds to k_b = 0.12 1/s
n	6		
K	30	nM	

δ	0.2	
E. coli cell length	2	μm
Total simulation time	3600	s

References

1. Ramamurthi KS, Losick R. Negative membrane curvature as a cue for subcellular localization of a bacterial protein. *Proc. Natl Acad. Sci. USA* **106**, 13541-13545 (2009).

2. Lenarcic R, et al. Localisation of DivIVA by targeting to negatively curved membranes. *Embo J.* **28**, 2272-2282 (2009).

3. Muchova K, et al. Oligomerization of the Bacillus subtilis division protein DivIVA. *Microbiology* **148**, 807-813 (2002).

4. http://book.bionumbers.org.

5. The kinetics of a constant production and linear degradation can be mathematically solved with exponential time functions, with the degradation rate as the exponential-decay rate. For example, if there were no diffusion nor membrane-binding for u, the time-dependence for u would have been $u(t) = u_{ss}+(u_0-u_{ss})\exp(-k_d t)$, with u_0 as its arbitrary initial concentration, and $u_{ss}(\equiv k_p/k_d)$ is the steady-state concentration.

6. Strogatz, S. H. Nonlinear Dynamics and Chaos: With Applications to Physics, Biology, Chemistry, and Engineering. *Avalon Publishing* (2014).
