# Supplementary material for: Construction of intracellular asymmetry and asymmetric division in Escherichia coli
Source: Nat Commun. 2021 Feb 9;12:888. doi: 10.1038/s41467-021-21135-1 (PMC7873278; doi:10.1038/s41467-021-21135-1)
Supplement: Supplementary file 2 — Reporting Summary [file 41467_2021_21135_MOESM2_ESM.pdf]

nature research

Corresponding author(s): Hsiao-Chun Huang

Last updated by author(s): Dec 4, 2020

Reporting Summary

Nature Research wishes to improve the reproducibility of the work that we publish. This form provides structure for consistency and transparency in reporting. For further information on Nature Research policies, see our Editorial Policies and the Editorial Policy Checklist.

Statistics

For all statistical analyses, confirm that the following items are present in the figure legend, table legend, main text, or Methods section.

n/a	Confirmed	
☐	☒	The exact sample size (*n*) for each experimental group/condition, given as a discrete number and unit of measurement
☐	☒	A statement on whether measurements were taken from distinct samples or whether the same sample was measured repeatedly
☐	☒	The statistical test(s) used AND whether they are one- or two-sided<br>*Only common tests should be described solely by name; describe more complex techniques in the Methods section.*
☒	☐	A description of all covariates tested
☒	☐	A description of any assumptions or corrections, such as tests of normality and adjustment for multiple comparisons
☐	☒	A full description of the statistical parameters including central tendency (e.g. means) or other basic estimates (e.g. regression coefficient) AND variation (e.g. standard deviation) or associated estimates of uncertainty (e.g. confidence intervals)
☐	☒	For null hypothesis testing, the test statistic (e.g. *F*, *t*, *r*) with confidence intervals, effect sizes, degrees of freedom and *P* value noted<br>*Give P values as exact values whenever suitable.*
☒	☐	For Bayesian analysis, information on the choice of priors and Markov chain Monte Carlo settings
☒	☐	For hierarchical and complex designs, identification of the appropriate level for tests and full reporting of outcomes
☐	☒	Estimates of effect sizes (e.g. Cohen's *d*, Pearson's *r*), indicating how they were calculated

Our web collection on statistics for biologists contains articles on many of the points above.

Software and code

Policy information about availability of computer code

Data collection	ZEISS ZEN 2 (blue edition) microscope software was used for image acquisition.
Data analysis	Outlines and fluorescence intensity profiles of individual cells were extracted from cell lists obtained from Oufti (MATLAB runtime version 8.4; Compiled version: Aug. 25, 2015), and subjected to customized analysis programs written in Python (Version: 3.7.4). ISS VistaVision software (Version: 4.2.171.0) was used to obtain diffusion coefficients for FCS measurements.<br><br>The custom code for polarity analysis is available at https://github.com/dwgoblue/SynPolarityAnalysis (DOI: 10.5281/zenodo.4297656); modeling code is available at https://github.com/cherrihsu/asymmetrycells (DOI: 10.5281/zenodo.4305098).

For manuscripts utilizing custom algorithms or software that are central to the research but not yet described in published literature, software must be made available to editors and reviewers. We strongly encourage code deposition in a community repository (e.g. GitHub). See the Nature Research guidelines for submitting code & software for further information.

Data

Policy information about availability of data

All manuscripts must include a data availability statement. This statement should provide the following information, where applicable:

- Accession codes, unique identifiers, or web links for publicly available datasets
- A list of figures that have associated raw data
- A description of any restrictions on data availability

Source data underlying Figs. 1b, 1d, 2b, 2d, 2f, 3c-d, 4f-g, 5a-c, 6a-b, 7a-b, 7d and Supplementary Figs. 1, 6-7, 10-11, 12c-e, 13a-b, 14, 15b-c are provided as a Source Data file. All other data and materials from this work are available from the corresponding author upon reasonable request.

Field-specific reporting

Please select the one below that is the best fit for your research. If you are not sure, read the appropriate sections before making your selection.

- [x] Life sciences
- [ ] Behavioural & social sciences
- [ ] Ecological, evolutionary & environmental sciences

For a reference copy of the document with all sections, see nature.com/documents/nr-reporting-summary-flat.pdf

Life sciences study design

All studies must disclose on these points even when the disclosure is negative.

Sample size	FCS measurements were performed on 3 independently prepared samples for two conditions, and data from total of either 23 or 24 cells were analyzed and reported. Such sample size was found to result in p-value of <0.0001 (as indicated in Fig. 5b) between the two tested conditions and therefore justify the sample size is sufficient. For polarity, intracellular asymmetry and cell division analysis, at least 60 cells were analyzed for each condition. Such sample size again resulted in p-value of <0.0001 in almost all comparisons (one exception: Supplementary Fig. 12d, with a p-value of 0.003004).
Data exclusions	For conditions with PopZ, only cells with PopZ unipolar foci (consecutive 1's occupying less than 1/3 of the vector after automatic image thresholding using Otsu's method) were selected in the analysis. This criteria has not been pre-established; it has been very useful in automatic exclusion of cells with diffused or bipolar PopZ (due to variations in PopZ expression level) in this study.
Replication	All experiments were performed with at least three biologically independent samples. All attempts at replication were successful.
Randomization	Selection of colony after transformation and field of view in image acquisition were performed at random.
Blinding	Image profiles extracted from oufti were subjected to automatic analysis programs written in Python.

Reporting for specific materials, systems and methods

We require information from authors about some types of materials, experimental systems and methods used in many studies. Here, indicate whether each material, system or method listed is relevant to your study. If you are not sure if a list item applies to your research, read the appropriate section before selecting a response.

Materials & experimental systems

n/a	Involved in the study	
☐	☒	Antibodies
☒	☐	Eukaryotic cell lines
☒	☐	Palaeontology and archaeology
☒	☐	Animals and other organisms
☒	☐	Human research participants
☒	☐	Clinical data
☒	☐	Dual use research of concern

Methods

n/a	Involved in the study	
☒	☐	ChIP-seq
☒	☐	Flow cytometry
☒	☐	MRI-based neuroimaging

Antibodies

Antibodies used	anti-AmpC antibody (cat. no. MBS310846; MyBioSource, Inc.); HRP-conjugated secondary antibody (anti-mouse; cat. no. NEF822001EA; PerkinElmer)
Validation	anti-AmpC antibody: validated in ELISA and Western Blot. Manufacturer's website: https://www.mybiosource.com/monoclonal-ampc-antibody/beta-lactamase/310846
