# Supplementary material for: Construction of intracellular asymmetry and asymmetric division in Escherichia coli
Source: Nat Commun. 2021 Feb 9;12:888. doi: 10.1038/s41467-021-21135-1 (PMC7873278; doi:10.1038/s41467-021-21135-1)
Supplement: Supplementary file 3 — Description of Additional Supplementary Files [file 41467_2021_21135_MOESM3_ESM.pdf]

Description of Additional Supplementary Files

Title: Supplementary Data 1
Description: Genetic constructs used in this study

Title: Supplementary Data 2
Description: Primers used in this study
